# Factors affecting mortality of hospitalized chest trauma patients in United Arab Emirates

**DOI:** 10.1186/1749-8090-8-57

**Published:** 2013-03-30

**Authors:** Essa M AlEassa, Mariam J Al-Marashda, Amgad Elsherif, Hani O Eid, Fikri M Abu-Zidan

**Affiliations:** 1 Trauma Group, Department of Surgery, Faculty of Medicine and Health Sciences, UAE University, P.O. Box 17666, Al-Ain, United Arab Emirates; 2 Cardiothoracic Surgery Division, Department of Surgery, Tawam Hospital, Al-Ain, United Arab Emirates; 3 Department of Surgery, Al-Ain Hospital, Al-Ain, United Arab Emirates

**Keywords:** Chest, Thorax, Trauma, Injury, Mortality

## Abstract

**Background:**

Predictors of mortality of chest trauma vary globally. We aimed to define factors affecting mortality of hospitalized chest trauma patients in Al-Ain City, United Arab Emirates.

**Methods:**

The data of Al-Ain Hospital Trauma Registry were prospectively collected over a period of three years. Patients with chest trauma who were admitted for more than 24 hours in Al-Ain Hospital or who died after arrival to the hospital were included in the study. Univariate analysis was used to compare patients who died and those who survived. Gender, age, nationality, mechanism of injury, systolic blood pressure and GCS on arrival, the need for ventilatory support, presence of head injury, AIS for the chest and head, presence of injuries outside the chest, and ISS were studied. Significant factors were then entered into a backward stepwise likelihood ratio logistic regression model.

**Results:**

474 patients having a median (range) age of 35 (1–90) years were studied. 90% were males and 18% were UAE citizens. The main mechanism of injury was road traffic collisions (66%) followed by falls (23.4%). Penetrating trauma occurred in 4 patients (0.8%). 88 patients (18.6%) were admitted to the ICU. The median (range) ISS was 5 (1–43). 173 patients (36.5%) had isolated chest injury. Overall mortality rate was 7.2%. Mortality was significantly increased by low GCS (p < 0.0001), high ISS (p = 0.025), and low systolic blood pressure on arrival (p = 0.027).

**Conclusion:**

Chest trauma is associated with a significant mortality in Al-Ain City. This was significantly related to the severity of head injury, injury severity score, and hypotension on arrival.

## Background

Injury is a leading cause of death worldwide and is the second cause of death in the United Arab Emirates (UAE). The mortality rate of patients having chest trauma ranges between 4% and 20% worldwide [1]. The pattern of chest trauma and its clinical presentation vary between different countries [2]. Consequently, predictors of mortality vary in different settings. When chest trauma is not life threatening, management decisions become less clear [1]. Clinical presentations alone have a low predictive value of mortality and may not be enough to guide the decision making process [3].

Studies on predictors of mortality of chest trauma are limited. Majority studied specific age groups like children and elderly, or studied specific chest injuries. Predictors of mortality of chest trauma have been defined in different settings [1]. Significant factors included the age, presence of flail chest, co-morbidities, number of fractured ribs, Glasgow Coma Scale (GCS), Injury Severity Score (ISS) and quality of pre-hospital care [1,4-7]. There are no similar studies from the Middle East. We aimed to define the factors affecting mortality of hospitalized chest trauma patients in Al-Ain, UAE.

## Methods

Patients with chest trauma who were admitted for more than 24 hours in Al-Ain Hospital or who died after arrival to the hospital were included in the study. Al-Ain Hospital is one of the two major public hospitals in Al-Ain City. The hospital has a catchment population of more than 350 000 inhabitants. During the study period, Al-Ain Hospital treated 80% of the trauma patients of Al-Ain City.

Data for patients were retrieved from Al-Ain Hospital Trauma Registry and were collected prospectively over 3 years (March 2003 to march 2006). The details of the development of the registry and its content were described elsewhere [8,9]. Injury Severity Scale (ISS) was calculated manually using the Abbreviated Injury Scale handbook [10]. Data which were required to score the AIS and ISS were available for all patients except for one who died in the Emergency room. The Local Ethics Committee of Al-Ain Health District Area has approved data collection for the Trauma Registry. All patients admitted to Al-Ain Hospital or their caregivers signed a general consent form giving permission to use their anonymous data for research and audit.

Demography of patients, mechanism of injury, systolic blood pressure and Glasgow Coma Scale (GCS) on arrival, the need for ventilation, ISS, presence of head or chest injury, Abbreviated Injury Scale (AIS) for both the chest and head injuries, and mortality were analyzed.

Statistical analysis included Fisher’s exact test for categorical data for two independent groups, Mann–Whitney U-test for continuous or ordinal data for two independent groups. Univariate analysis was used to compare patients who survived and those who died. Significant factors were then entered into a backward stepwise likelihood ratio logistic regression. A p value of ≤ 0.05 was considered significant. Data were analyzed with PASW Statistics 18, SPSS Inc, USA.

## Results

474 patients were studied, 425 (90%) were males. Median (range) age was 35 (1–90) years. Eighty six patients (18.1%) were UAE nationals. The most common mechanism of injury was road traffic collision (RTC) in 313 patients (66%) followed by falls in 111 patients (23.4%). Penetrating trauma occurred in only 4 patients (0.8%), all were stab wounds and all survived. 304 (64.1%) patients were injured in the street or highway, 90 (19%) at work place, and 45 (9.5%) at home. Two hundred sixty five (55.9%) patients arrived at hospital by ambulance.

One hundred and seventy three (36.5%) patients sustained isolated chest injury while 301 (63.5%) patients had associated injuries in other regions. The most common associated injured regions were the head (27.4%), lower limbs (25.1%), and upper limbs (24.9%) (Table 1). The median (range) ISS was 5 (1–43). The median (range) GCS was 13 (3–15). Eighty eight patients (18.6%) were admitted to the ICU, 28 of them died (32%). Overall, 34 patients died (7.2%).

**Table 1:** Distribution of associated extrathoracic injuries of chest trauma patients in Al-Ain Hospital (2003–2006), n = 474

**Body region**	**Number (%)**
Head	130 (27.4%)
Neck	16 (3.4%)
Abdomen	65 (13.7%)
Spine	56 (11.8%)
Upper extremity	118 (24.9%)
Lower extremity	119 (25.1%)

Univariate analysis has shown that patients who died had significantly lower GCS and systolic blood pressure on arrival to hospital. They also had significantly higher ISS and AIS of both the head and chest, and had a higher need for mechanical ventilation. Those who died had significantly higher percentage of head injury (50%). Males had significantly higher mortality compared with females; 7.5% compared with 4.1% respectively (p < 0.002 Fisher’s exact test). Road traffic collision had the highest mortality among the different mechanisms of injury (9.6%) (Table 2).

**Table 2:** Univariate analysis comparing chest trauma patients who survived and those who died

**Variable**	**Survived (%) n = 440**	**Non-survivor (%) n = 34**	**p value**
Age years	35 (1–90)	32 (4–75)	0.97
Gender			0.002
Male	393 (82.9%)	32 (6.8%)	
Female	47 (9.9%)	2 (0.4%)	
Nationality			0.4
UAE	79 (16.7%)	7 (1.5%)	
Non UAE	357 (75.3%)	26 (5.5%)	
Systolic blood pressure mmHg	133 (78–222)	99 (56–215)	0.002
GCS	15 (3–15)	5 (3–15)	<0.0001
ISS	4 (1–41)	25 (8–43)	<0.0001
Highest AIS head	1 (1–4)	3 (1–4)	<0.0001
Highest AIS chest	1 (1–4)	3 (1–5)	<0.0001
Mechanism			0.003
Road traffic collision	283 (59.7%)	30 (6.3%)	
Fall	110 (23.2%)	1 (0.2%)	
Others^**a**^	47 (9.9%)	3 (0.6%)	
Ventilated	35 (7.4%)	25 (5.3%)	<0.0001
Head injury	107 (22.6%)	17 (3.6%)	0.002
Associated injuries	278 (58.6%)	23 (4.9%)	0.71

Data presented as median (range) or numbers (%) as appropriate. Percentages of the overall number of patients (n = 474).

p value = Mann Whitney U test or Fisher’s exact test as appropriate.

a: Penetrating trauma occurred in 4 patients, all were stab wounds and all survived.

The logistic regression model has shown that the significant factors that predicted mortality were low GCS (p < 0.0001), low systolic blood pressure on presentation (p=0.027) and high ISS (p < 0.025) (Table 3).

**Table 3:** Backward logistic regression model defining factors affecting mortality of patients having chest trauma

**Variable**	**Estimate**	**SE**	**Wald test**	**p value**	**Odds ratio**	**95% CI of OR**
GCS	- 0.344	0.09	16.08	< 0.0001	0.71	0.6-0.84
ISS	0.08	0.04	5.04	0.025	1.09	1.01-1.17
Systolic blood pressure	−0.03	0.01	4.86	0.027	0.98	0.95-0.997

**Figure 1:**
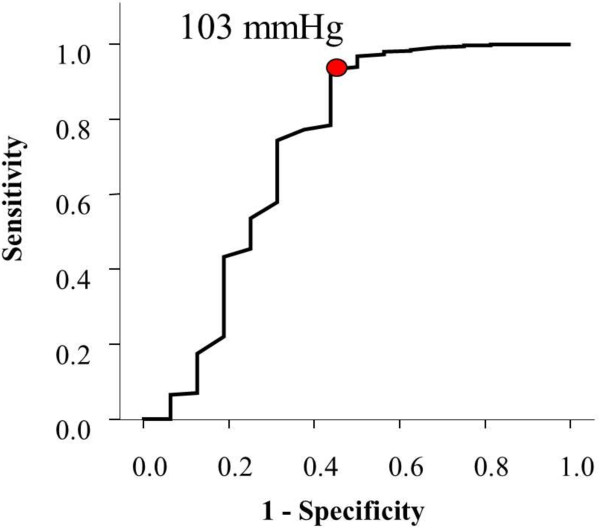
Receiver Operating Characteristic curve for the cutoff point of systolic blood pressure that best predicts mortality.

**Figure 2:**
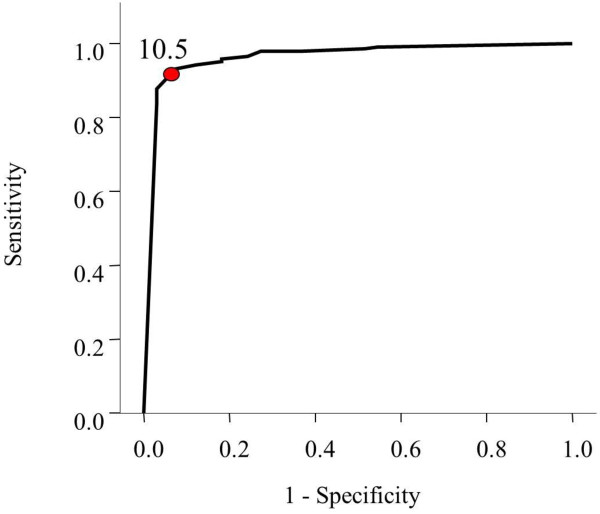
Receiver Operating Characteristic curve for the cutoff point of GCS that best predicts mortality.

**Figure 3:**
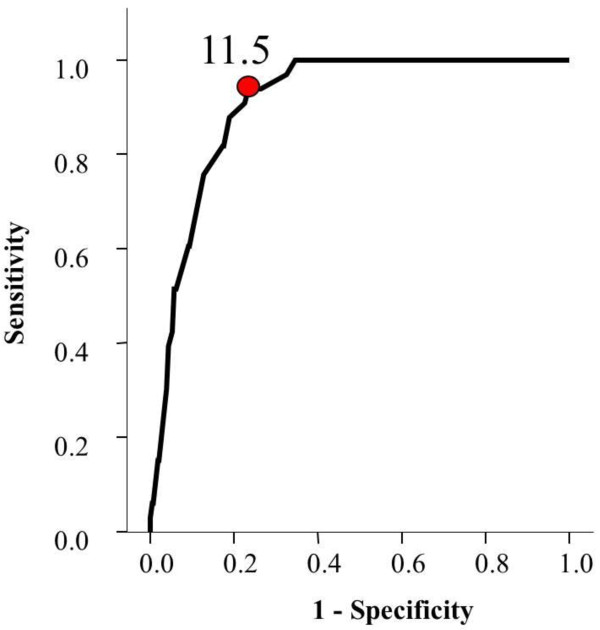
Receiver Operating Characteristic curve for the cutoff point of ISS that best predicts mortality.

The Receiver Operating Characteristic (ROC) curve has shown that best cut off points for predictors of mortality were systolic blood pressure of less than 103 mmHg on presentation (Figure 1), GCS of less than 10.5 (Figure 2) and ISS of more than 11.5 (Figure 3).

## Discussion

Our study has shown that significant predictors for mortality of chest trauma patients were low GCS, hypotension on presentation and increased severity of injury. Old age was reported as a predictor of mortality for chest trauma [2,4,5,11]. Liman et al. have shown that an age of more than 60 years significantly increased mortality [5]. Patients’ demographics (age, gender and nationality) did not predict mortality in our logistic regression model. The UAE population is a very young population composed mainly of male workers [12,13]. Elderly expatriate patients prefer to retire at their homeland. Having small number of elderly patients in our study may have resulted in the non significant effect of age on mortality.

Majority of chest trauma in our community is blunt in nature. There is less than 1% penetrating trauma. The strong law enforcement in our community contributes to the reduction of domestic violence because aggressors may be deported to their homeland if they cause physical injury to others.

Univariate analysis has shown that the mechanism of injury, mechanical ventilation, and admission to the ICU, head and chest AIS, ISS, GCS and systolic blood pressure on arrival to hospital were significantly different between those who died and those who survived. The multivariate logistic regression model excluded all these factors except ISS, GCS and systolic blood pressure as significant predictors of mortality.

Our cut off values for prediction were systolic blood pressure of less than 103 mmHg, GCS lower than 10.5 and an ISS score higher than 11.5. In contrast, Emircan et al. found the same significant factors with different cut off values. These were hypotension (in general), GCS lower than 13 and an ISS higher than 22 [2]. Patients having a lower ISS, compared with Emircan et al., died in our setting. We think that this occurred because our prehospital care is relatively not well developed. Lack of pre-hospital care increases mortality [14] while improved pre-hospital care reduced mortality in chest trauma patients [7].

RTC is the most common mechanism of chest trauma [5]. Abbas et al. have described the biomechanics of chest trauma in RTC [15]. They defined three mechanisms of injury occurring in front impact collisions of unrestrained drivers. This includes the impact of the chest to steering wheel, and the head injury to the wind shield. A recent prospective study has shown that more than 80% of RTC patients in the UAE were unrestrained [16]. This finding explains the high frequency and severity of head injury in our study. Seatbelt usage reduces severity of head injury [17]. Emircan et al. highlighted that the mechanism of injury is a significant predictor of mortality. The most common associated extra-thoracic injury in our study was to the head; which reflects the low usage of seatbelts [18].

Similar to others, our study has shown that chest injuries associated with extra-thoracic injuries were more common than isolated chest injuries [2,5]. In contrast, Demirhan et al. reported the opposite [6]. This can be explained by selection bias. Presence of associated extrathoracic injuries, especially abdominal injuries increases the mortality [2,11]. Splenic and hepatic injuries increase the risk of death by threefold [8]. In contrast, the type of intra-thoracic injury per se was not a predictor for mortality in chest trauma patients [7].

### Limitations of the study

We have to note that our study includes only patients who were admitted in the hospital for more than 24 hours or those who died in the Emergency room. It does not include patients with more severe injuries who died before arriving to the hospital, those with minor injuries who were treated in the Emergency Department, and those who were hospitalized for less than 24 hours. Pre-hospital care details are not available in our data. This is a major limitation of our study because pre-hospital care can be a major predictor of mortality.

Furthermore, our data represents the period before 2006 which may not exactly reflect what happens at present. Establishing the Trauma Registry of Al-Ain Hospital was a specific limited research project supported by the UAE University. Nevertheless, we, think that the factors affecting mortality of chest trauma did not change since then. Despite the fact that pre-hospital care has improved, the observed severity of head injury in our setting is still high because of the very low compliance of seatbelt usage [16].

Furthermore, other important predictors for chest trauma mortality which were reported in the literature are missing in our study. This includes the number of fractured ribs, the presence of co-morbidities, and the use of seatbelts. A strong correlation between the number of fractured ribs and mortality was demonstrated by several authors [5,19,20]. Presence of co-morbidities was also a significant predictor of chest trauma mortality [7]. Data on co-morbidities were not available in our study. Nevertheless, we anticipate that this factor was not important in our young population.

### Statistical considerations

There are two cautions that have to be addressed in this analysis, the missing data and the linearity of the model. We assumed that missing data of a variable were random and were not replaced by the average of that variable. Since there is a risk that some variables were eliminated by the backward logistic regression model because of missing data, a direct logistic regression model for the significant factors was redone. The significant factors were the same supporting our assumption that missing data were random.

To address the linearity concern, we have entered ISS^2^ and (systolic blood pressure)^2^ to the direct logistic model and these new variables turned out to be non significant. Furthermore, we categorized GCS into three categories; severe head injury (GCS=3-8), moderate head injury (GCS=9-13), and mild head injury (GCS=14-15) and redone the direct logistic regression model. The odds ratio of death of severe head injury was 61.4 (95% CI, 6–630) compared with mild head injury. The odds ratio of death of moderate head injury was 19.6 (95% CI, 1.92-200) compared with mild head injury. We could have used a more robust test for linearity like the fractional polynomial analysis instead of the methods used. Nevertheless, it would have been difficult to prove the exact linearity of the model because of the small sample size. We think that the above findings justify the assumption of approximate linearity in our logistic regression model given that the results are interpreted cautiously.

Finally, it is recommended to have ten events per each variable entered in the logistic regression model [21]. We have entered eight variables having 34 events (deaths) into the model. This ideally needs 80 events for proper statistical analysis. Nevertheless, other methodologists [22] have recently argued that “the rule of ten” should be relaxed. We think that our results are still valid if interpreted cautiously [21].

## Conclusions

Road traffic collision is the main cause of chest trauma in Al-Ain City, United Arab Emirates. The mortality of patients presenting with chest trauma is high in our setting (7.2%). This was significantly related to the severity of injury and hypotension on arrival. Extra-thoracic injuries are the main cause of death in chest trauma patients. Improvement in the pre-hospital care and enforcement of seatbelt compliance may reduce this mortality.

## Competing interest

There is no conflict of interest declared by all authors.

## Authors’ contributions

EMA helped in the idea, collected the literature, wrote the manuscript and approved the final version of the manuscript. MJA helped in the idea, analyzed the data, and approved the final version of the manuscript. AE helped in the idea, critically read the paper, and approved the final version of the manuscript. HOE helped in the idea, collected the data, critically read the paper, and approved the final version of the manuscript. FAZ had the idea, defined the research protocol, assured the quality of data collected, did the advanced statistical analysis, helped draft the first version of the paper, repeatedly edited it, answered the concerns of the reviewers, revised the manuscript, and approved its final version.
